# Obesity and Metabolic Phenotypes (Metabolically Healthy and Unhealthy Variants) Are Significantly Associated with Prevalence of Elevated C-Reactive Protein and Hepatic Steatosis in a Large Healthy Brazilian Population

**DOI:** 10.1155/2015/178526

**Published:** 2015-03-09

**Authors:** Sameer Shaharyar, Lara L. Roberson, Omar Jamal, Adnan Younus, Michael J. Blaha, Shozab S. Ali, Kenneth Zide, Arthur A. Agatston, Roger S. Blumenthal, Raquel D. Conceição, Raul D. Santos, Khurram Nasir

**Affiliations:** ^1^Aventura Hospital and Medical Center, Aventura, FL 33180, USA; ^2^Center for Prevention and Wellness Research, Baptist Health South Florida, Miami, FL 33139, USA; ^3^Department of Epidemiology, Robert Stempel College of Public Health, Florida International University, Miami, FL 33199, USA; ^4^Johns Hopkins Ciccarone Center for Preventive Cardiology, Johns Hopkins University, Baltimore, MD 21287, USA; ^5^Preventive Medicine Center, Avenida Albert Einstein 627/701, 05652-900 Morumbi, SP, Brazil; ^6^Heart Institute (InCor) University of São Paulo Medical School Hospital & Preventive Medicine Center, Hospital Israelita Albert Einstein, 05652-900 São Paulo, SP, Brazil; ^7^Herbert Wertheim College of Medicine, Florida International University, Miami, FL 33199, USA

## Abstract

*Background*. Among the obese, the so-called metabolically healthy obese (MHO) phenotype is thought to confer a lower CVD risk as compared to obesity with typical associated metabolic changes. The present study aims to determine the relationship of different subtypes of obesity with inflammatory-cardiometabolic abnormalities. *Methods*. We evaluated 5,519 healthy, Brazilian subjects (43 ± 10 years, 78% males), free of known cardiovascular disease. Those with <2 metabolic risk factors (MRF) were considered metabolically healthy, and those with BMI ≥ 25 kg/m^2^ and/or waist circumference meeting NCEP criteria for metabolic syndrome as overweight/obese (OW). High sensitivity C reactive protein (hsCRP) was measured to assess underlying inflammation and hepatic steatosis (HS) was determined via abdominal ultrasound. *Results*. Overall, 40% of OW individuals were metabolically healthy, and 12% normal-weight had ≥2 MRF. The prevalence of elevated CRP (≥3 mg/dL) and HS in MHO versus normal weight metabolically healthy group was 22% versus 12%, and 40% versus 8% respectively (*P* < 0.001). Both MHO individuals and metabolically unhealthy normal weight (MUNW) phenotypes were associated with elevated hsCRP and HS. *Conclusion*. Our study suggests that MHO and MUNW phenotypes may not be benign and physicians should strive to treat individuals in these subgroups to reverse these conditions.

## 1. Introduction

Obesity has been clearly linked with dyslipidemia, hypertension, and insulin resistance; however not every obese patient has these metabolic derangements [1]. A new phenotype has been identified within the obese population who do not have the typical milieu of obesity-related metabolic disturbances. These patients are termed “metabolically healthy obese” (MHO) [2–4] and were initially thought to have little or no increased risk of cardiovascular disease (CVD) or cancer when compared to metabolically healthy normal weight controls (MHNW).

Published literature is in disagreement regarding the relative risk of disease in the MHO population. Several epidemiologic studies have shown that MHO participants are at similar or decreased risk of developing CVD compared with MHNW participants [5–11]. Studies with longer follow-up periods (>15 years) have demonstrated that MHO individuals were at an increased risk of major CVD events as compared to MHNW individuals [12, 13]. However, there are very few studies examining the relationship between the MHO phenotype and subclinical markers of CVD.

In addition, another subgroup of obesity has been defined, variously referred to as “metabolically obese but normal weight” or “metabolically unhealthy normal weight” (MUNW) [14]. This group, in contrast to the MHO group, displays the typical obesity related metabolic disturbances of hyperinsulinemia, premature insulin resistance, hypertriglyceridemia, and possibly elevated risk of developing diabetes and CVD [14]. There are few studies examining vascular inflammation or hepatic steatosis (HS) explicitly in this population.

The aim of this study was to explore the association among MHO, MUNW, and markers of subclinical CVD burden as assessed by high sensitivity C-reactive protein (CRP) and hepatic steatosis (HS) in individuals without overt CVD. We sought to identify whether MHO and MUNW individuals had similar risk profiles to MHNW individuals and were therefore at a lower risk than metabolically unhealthy obese (MUHO) phenotypes.

## 2. Methods

This cross sectional study was conducted among asymptomatic individuals without a history of coronary heart disease or CVD events presenting to the Preventive Medical Center of the Hospital Israelita Albert Einstein, Sao Paulo, Brazil, as part of a mandatory occupational health evaluation. The study was approved by the Hospital's Institutional Review Board. A total of 6461 healthy subjects were evaluated. Information collected included demographic details, self-reported history of medical conditions, and use of medication including antihypertensives, antidiabetics, and lipid lowering medications. Anthropometric measurements included weight, height and waist circumference.

Fasting blood samples were obtained for plasma lipids including high density lipoprotein cholesterol (HDL), low density lipoprotein cholesterol (LDL), triglycerides (TG), blood glucose, liver enzymes (AST, ALT, and GGT), and high-sensitivity C-reactive protein (hs-CRP). Elevated hs-CRP was defined as ≥3 mg/L and was determined by immunonephelometry (Dade-Behring). Presence of HS was assessed by abdominal ultrasound [15]. All tests were performed at the central laboratory of the Albert Einstein Hospital. Metabolic risk factors (MRFs) were defined according to updated NCEP ATP III criteria as follows: triglycerides ≥ 150 mg/dL; HDL ≤ 40 mg/dL in males or HDL ≤ 50 mg/dL in females, blood pressure ≥ 130/85 mm Hg or on antihypertensives, and fasting glucose ≥ 100 mg/dL or on antidiabetic medications. Those individuals with less than two MRFs were considered to be metabolically healthy and those with two or more MRFs were labeled metabolically unhealthy [16]. Obesity was defined as BMI > 25 kg/m^2^ and/or waist circumference > 40 inches in males and >35 inches in females. Four groups were identified: metabolically healthy normal weight (MHNW), metabolically unhealthy normal weight (MUNW), metabolically healthy obese (MHO), and metabolically unhealthy obese (MUHO).

### 2.1. Statistical Analysis

All statistical analyses were performed with STATA Statistical Software, Release 12.0 (Stata Corporation Inc., College Station, TX). After removing subjects with missing data, 5,519 participants were included in the analyses. Metabolically healthy normal weight individuals formed the referent group. Analysis of variance tests (ANOVA) and chi-square tests of independence were used for continuous and categorical variables, respectively. Logistic regression analysis was performed, adjusting for age, gender, physical activity, LDL, and current smoking status. A *P* value <0.05 was considered to be statistically significant.

## 3. Results

The majority of participants were male (78%). Surprisingly, 62% of the participants were found to be obese (BMI ≥ 25 kg/m^2^) and 37% of the participants were identified as being MHO.  Table 1 shows the baseline characteristics of the population selected for analysis, stratified according to metabolic status and presence of obesity. The most common phenotypes were MHO (37%) and MHNW (34%). MHO individuals tended to be older than the referent group but had a lower average age than the MUHO group (*P* < 0.001). Almost all variables assessed including determinants of metabolic syndrome and metabolic parameters (LDL, total cholesterol and liver enzymes) differed significantly across groups. The “higher risk” values were detected in the MUHO group, followed by the MHO, MUNW, and MHNW groups, respectively. The prevalence of hypertension, diabetes, and dyslipidemia was higher in the metabolically unhealthy groups as compared to the metabolically healthy phenotypes (Table 1).

Table 2 shows the prevalence for elevated hsCRP and hepatic steatosis among the four groups. The highest prevalence of elevated hsCRP and hepatic steatosis was observed in the MUHO group, followed by the MHO and MUNW groups. Although individuals with both metabolic abnormalities and obesity were at the highest risk of CRP and steatosis, the presence of metabolic abnormalities or obesity alone was associated with an almost 2-fold higher risk of vascular inflammation as well as a 3–5 fold risk of steatosis, respectively. In a subgroup analysis, MUNW was not associated with increased prevalence of hsCRP ≥3 in females. Apart from this difference, similar graded associations between MHO and MUHO were observed in both genders for both vascular inflammation and hepatic steatosis (Table 2), although with lower precision, that is, wider confidence intervals.

## 4. Discussion

The present study shows that MHO and MUNW groups have a higher prevalence of elevated hs-CRP levels and hepatic steatosis as compared to MHNW group. However, the highest prevalence of these markers was recorded in the MUHO group. This suggests that obesity per se in absence of metabolic risk factors is not entirely benign and is in fact associated with subclinical vascular inflammation. In addition, we also document a higher-than-expected prevalence of obesity in otherwise healthy Brazilian subjects, possibly due to the selection bias of government employees.

Adipose tissue is increasingly being recognized as an endocrine organ as well as a highly metabolically active tissue, responsible for production of a large number of cytokines including TNF-*α* and IL-6 [17, 18]. These adipocytokines are in turn believed to contribute to the metabolic disturbances associated with obesity. This understanding suggests that obesity may result in a proinflammatory metabolic state and may explain the higher prevalence of elevated inflammatory markers in our MHO population. The MHO population would therefore be expected to have greater endothelial dysfunction and subclinical atherosclerotic disease as compared to MHNW [19–21]. A recent study examined the natural history of the MHO phenotype and found that over half of subjects progressed to frank metabolic syndrome over a 10 year period [22]. Our results, may reflect this phenomenon, as the MHO population is younger than that of the MUHO cohort, thus the MHO individual may be a harbinger of the future MUHO patient. A recent review by our team demonstrated that the MHO phenotype was linked with adverse CVD outcomes; however, systemic inflammation was not explicitly assessed in the majority of the studies reviewed [23]. The few that did examine systemic inflammation failed to demonstrate a relationship with CVD outcomes. This does not, however, disprove the present hypothesis of a proinflammatory state being present in the MHO phenotype, although it does raise questions about the potential clinical implications.

Also, MUNW phenotype is thought to represent a fundamental dysfunction of metabolism that is thought to be closely related to obesity, but is not uncommon among those with normal weight. Seppälä-Lindroos et al. also documented higher liver fat concentration and derangement of insulin and lipid metabolism in MUNW individuals [24], suggesting that the MUNW should be considered a high risk phenotype. The present study is in agreement, in that an overwhelming number of subjects who were classified as metabolically unhealthy suffered from dyslipidemia, which is a classical and well established risk factor for cardiovascular disease. Unfortunately, the relatively small number of individuals classified as MUNW in our study (*n* = 259), limits the statistical power of subgroup analysis exploring the possible additive effect of hypertension and dyslipidemia on promoting vascular inflammation.

Our findings should be considered in the light of a few limitations. Firstly, we employed surrogate markers for cardiovascular disease; the true risk in this population may thus be lower than our estimates suggest; however, the magnitude of our results makes our findings relatively robust. Secondly, this is a cross sectional study and therefore can only demonstrate associations and not causation. Consequently, longitudinal studies are needed in order to assess the relationship between these phenotypes, subclinical disease markers and hard outcomes in order to accurately determine this population's true risk. While a growing body of literature has suggested that this population may be at higher risk [23], no consensus has yet been reached.

In conclusion, our study indicates that both MHO and MUNW phenotypes are associated with higher burden of inflammation when compared to MHNW subjects, albeit not as strongly as the MUHO phenotype. Certainly, future research should be devoted to refine CVD risk in these newly appreciated subpopulations, so that physicians can effectively identify individuals at risk for tailored therapeutic and preventive interventions.

## Figures and Tables

**Table 1:** Baseline population characteristics according to metabolic status and obesity.

	Metabolically fealthy normal weight (*n* = 1864)	Metabolically unhealthy normal weight (*n* = 259)	Metabolically healthy overweight/obese (*n* = 2025)	Metabolically unhealthy overweight/obese (*n* = 1371)	*P* values (between group differences)
Age (yrs)	41 ± 9	46 ± 10	43 ± 9	47 ± 10	<0.001
Male *n* (%)	1100 (59%)	209 (80%)	1731 (85%)	1264 (92%)	<0.001
Metabolic syndrome determinants		
BMI (kg/m^2^)	22.58 ± 1.73	23.47 ± 1.25	27.97 ± 2.85	29.70 ± 3.74	<0.001
Systolic BP (mm Hg)	112 ± 10	121 ± 13	119 ± 10	128 ± 13	<0.001
Diastolic BP (mm Hg)	73 ± 7	77 ± 8	77 ± 7	82 ± 7	<0.001
Glucose (mg/dL)	85 ± 8	90 ± 9	89 ± 8	94 ± 14	<0.001
Triglycerides (mg/dL)^*^	89 (69–117)	183 (145–236)	107 (82–137)	189 (154–245)	<0.001
HDL (mg/dL)	55 ± 14	40 ± 9	49 ± 11	39 ± 9	<0.001
Hypertension	139 (7%)	116 (45%)	397 (20%)	919 (67%)	<0.001
Diabetes/↑FPG	7 (<1%)	12 (5%)	13 (<1%)	183 (13%)	<0.001
Dyslipidemia	462 (25%)	256 (98%)	700 (35%)	1344 (98%)	<0.001
Anthropometric data					
Waist (cm)	81 ± 7	86 ± 6	96 ± 9	102 ± 10	<0.001
Waist hip ratio	0.84 ± 0.07	0.90 ± 0.06	0.92 ± 0.07	0.96 ± 0.06	<0.001
Other variables					
Total cholesterol (mg/dL)	199 ± 33	208 ± 41	208 ± 37	214 ± 41	<0.001
Non-HDL (mg/dL)	143 ± 35	168 ± 42	159 ± 37	175 ± 40	<0.001
LDL (mg/dL)	124 ± 31	129 ± 35	136 ± 33	134 ± 35	<0.001
AST (IU/L)	28 ± 10	30 ± 9	32 ± 11	34 ± 12	<0.001
ALT (IU/L)	29 ± 15	36 ± 16	40 ± 22	46 ± 24	<0.001
GGT (IU/L)	29 ± 21	39 ± 26	41 ± 37	52 ± 47	<0.001
hs-CRP^*^	0.8 (0.5–1.6)	1.1 (0.6–2.2)	1.4 (0.8–2.7)	1.7 (1.0–3.0)	<0.001
Presence of steatosis *n* (%)	152 (8%)	70 (27%)	805 (40%)	952 (70%)	<0.001
Elevated CRP *n* (%)	223 (12%)	44 (17%)	452 (22%)	343 (25%)	<0.001

^*^Data expressed as median (interquartile range).

**Table 2:** Prevalence and odds ratios for vascular inflammation and hepatic steatosis across weight and metabolic risk factor based phenotypes.

Prevalence of Elevated hsCRP									
	Overall			Males only			Females only		
	*n* (%)	UOR	AOR	*n* (%)	UOR	AOR	*n* (%)	UOR	AOR
Metabolically healthy normal weight (referent)	223 (12%)	1	1	96 (9%)	1	1	127 (16%)	1	1
Metabolically unhealthy normal weight	44 (17%)	1.51 (1.06–2.14)	2.00 (1.31–3.06)	35 (17%)	2.10 (1.38–3.20)	1.96 (1.23–3.11)	9 (18%)	1.10 (0.52–2.32)^*^	1.24 (0.58–2.63)^*^
Metabolically healthy obese	452 (22%)	2.11 (1.78–2.52)	2.45 (1.92–3.12)	335 (19%)	2.51 (1.97–3.19)	2.52 (1.94–3.27)	117 (40%)	3.32 (2.45–4.48)	3.59 (2.63–4.90)
Metabolically unhealthy obese	343 (25%)	2.46 (2.04–2.96)	2.89 (2.25–3.73)	283 (22%)	3.02 (2.36–3.86)	2.93 (2.24–3.84)	60 (56%)	6.40 (4.18–9.81)	7.84 (4.95–12.41)

Prevalence of Hepatic Steatosis									
	Overall			Males Only			Females Only		
	*n* (%)	UOR	AOR	*n* (%)	UOR	AOR	*n* (%)	UOR	AOR

Metabolically healthy normal weight (referent)	152 (8%)	1	1	137 (13%)	1	1	15 (2%)	1	1
Metabolically unhealthy normal weight	70 (27%)	4.12 (2.99–5.68)	3.03 (2.12–4.32)	62 (30%)	2.94 (2.08–4.17)	2.60 (1.82–3.69)	8 (16%)	9.30 (3.7–23.15)	6.67 (2.59–17.18)
Metabolically healthy obese	805 (40%)	7.49 (6.20–9.05)	5.80 (4.72–7.13)	756 (71%)	5.50 (4.49–6.74)	5.30 (4.32–6.50)	49 (17%)	10.13 (5.58–18.40)	8.57 (4.67–15.73)
Metabolically unhealthy obese	952 (70%)	25.76 (21.04–31.54)	17.64 (14.14–22.01)	894 (71%)	17.03 (13.71–21.15)	15.18 (12.19–19.49)	58 (56%)	62.89 (33.08–119.56)	44.41 (22.81–86.49)

UOR: unadjusted odds ratio (95% confidence interval).

AOR: adjusted odds ratio (95% confidence interval), adjusted for age, gender, LDL, smoking history, and physical activity.

*n* (%) represents number and percentage of subjects with outcome of interest (steatosis or elevated hsCRP) among a specific phenotype (MHNW, MUNW, MHO or MUHO).

^*^ *P* value > 0.05. All other data has associated *P* < 0.05 unless otherwise noted.
